# Pore-Scale Modeling of Pore Structure Effects on P-Wave Scattering Attenuation in Dry Rocks

**DOI:** 10.1371/journal.pone.0126941

**Published:** 2015-05-11

**Authors:** Zizhen Wang, Ruihe Wang, Tianyang Li, Hao Qiu, Feifei Wang

**Affiliations:** 1 School of Petroleum Engineering, China University of Petroleum (Huadong), Qingdao, Shandong, People’s Republic of China; 2 College of Pipeline and Civil Engineering, China University of Petroleum (Huadong), Qingdao, Shandong, People’s Republic of China; University of Kansas, UNITED STATES

## Abstract

Underground rocks usually have complex pore system with a variety of pore types and a wide range of pore size. The effects of pore structure on elastic wave attenuation cannot be neglected. We investigated the pore structure effects on P-wave scattering attenuation in dry rocks by pore-scale modeling based on the wave theory and the similarity principle. Our modeling results indicate that pore size, pore shape (such as aspect ratio), and pore density are important factors influencing P-wave scattering attenuation in porous rocks, and can explain the variation of scattering attenuation at the same porosity. From the perspective of scattering attenuation, porous rocks can safely suit to the long wavelength assumption when the ratio of wavelength to pore size is larger than 15. Under the long wavelength condition, the scattering attenuation coefficient increases as a power function as the pore density increases, and it increases exponentially with the increase in aspect ratio. For a certain porosity, rocks with smaller aspect ratio and/or larger pore size have stronger scattering attenuation. When the pore aspect ratio is larger than 0.5, the variation of scattering attenuation at the same porosity is dominantly caused by pore size and almost independent of the pore aspect ratio. These results lay a foundation for pore structure inversion from elastic wave responses in porous rocks.

## Introduction

When elastic wave propagates through porous rocks, there will be geometry attenuation caused by energy dissipating due to wave front expansion, friction attenuation by relative sliding among grains, and scattering attenuation by heterogeneity [1]. Generally, the scattering attenuation is influenced by the inner micro-structure of rocks, such as cracks, pore structures and pore-fluid interactions [2–3]. Underground rocks, such as carbonate rocks, usually have varying pore systems with a variety of pore types and a wide range of pore size [4]. Therefore, the effects of pore structure on elastic wave attenuation cannot be neglected. Investigation of scattering attenuation of dry rocks is helpful to improve the reliability of fluid identification and quantitative fracture prediction in oil and gas reservoirs.

Researchers have conducted lots of work on mechanisms and rules of elastic wave attenuation in porous rocks. Theoretical studies mainly focus on coupling the solid-fluid movement under a stress wave, such as the Biot model [5], the Squirt and BISQ model [6–9], the partially saturated model [10–11] and the unified model [12–14]. Wei et al. [15] studied the influences of fluid properties, permeability and incidence angles on elastic wave attenuation based on the Biot model. Ba et al. [16], Sun et al. [17] and Amalokwu et al. [18] analyzed velocity dispersion and attenuation of P-waves in partially-saturated rocks. Some analytical modelings of elastic wave attenuation/dispersion based on microstructural models [19–22] have also been done to address this issue. These works generally consider the pore structure effects by parameters such as pore aspect ratio (*AR*, ratio of minor axis to major axis) and crack density. On the other hand, lots of experimental studies have also been carried out. Klimentos and McCann [23], Wang et al. [24] studied the P-wave attenuation in sandstones. Shi and Deng [25] analyzed the attenuation anisotropy of clay and shale by experiments under formation condition. Liu et al. [26] tested the ultrasonic wave attenuation characteristic of 28 carbonate cores from Sichuan basin, and analyzed the effects of gas saturation on the attenuation coefficient. Moreover, researchers [27–29] built seismic physical models based on the similarity principle of wave theory, and studied the attenuation responses of elastic waves to simple cracks and/or vugs combinations. However, the scattering attenuation caused by the heterogeneity of the solid matrix, pore structure and grains in dry rocks (excluding the effects of pore fluids) is still not fully understood.

Studying the relationship between rock properties and their microstructures by experiments is the most straightforward, but expensive and time-consuming, method. Numerical modeling is an important complement to experiments. Pore-scale modelings have proved to be practical and reliable for the modeling of rock electrical properties [30], failure and energy dissipation in rocks [31], fluid transportation [32], and acoustic attenuation in fractured media [33].

This paper attempts to quantify the pore structure effects on the P-wave scattering attenuation in dry rocks by pore-scale numerical modeling. Firstly, we built 2D numerical models of elastic wave propagation in porous rocks based on the wave theory and similarity principle, and described our data processing method of calculating scattering attenuation coefficient. Subsequently, we performed series of modelings with different pore structures by taking carbonate rocks as an example, and linked the scattering attenuation coefficient to pore structure parameters. Pore-scale modeling provides an effective way to explain the scattering attenuation variation at the same porosity for rocks with complex pore structures.

## Methods

### Similarity principle of wave theory

In order to make the elastic wave kinematic characteristic (such as amplitude and attenuation) of numerical modeling similar to that of real experiments, we need to build models meeting the requirements of geometrical similarity and kinematic similarity [34]. The elastic wave propagation in homogeneous elastic medium is controlled by [35]:

$$\mu \nabla^{2}u+\left(K+\frac{\mu }{3}\right)\text{grad}\left(\text{div}u\right)=\rho \frac{\partial^{2}u}{\partial t^{2}}$$ (1)

where *K* is the bulk modulus; *μ* is the shear modulus; *ρ* is the density of the medium; and ***u*** is the displacement vector. The wave equation in numerical modeling would be the same as that in experiments if:

$$\frac{\mu }{\mu_{\text{mod}}}=\frac{K}{K_{\text{mod}}}=\frac{\rho }{\rho_{\text{mod}}}$$ (2)

where parameters with the subscript mod are used in the numerical modeling. When Eq 2 is satisfied, the velocity of the modeling media is equal to the velocity of the actual rock. And according to geometrical similarity, it should be [34]:

$$\frac{f}{f_{\text{mod}}}=\frac{\lambda_{\text{mod}}}{\lambda }=\frac{l_{\text{mod}}}{l}=\frac{d_{\text{mod}}}{d}$$ (3)

where *f* and *λ* are respectively the frequency and wave length of the ultrasonic measurement in the lab; *d* is the average size of pores in the real core, and *l* is the length of the core. The wavelength of the ultrasonic test in the lab is much larger than the pore size, such that the long-wavelength Rayleigh scattering is appropriate for analyzing petrophysical lab results. The scattering attenuation coefficient *α*_s_ can be written as [2–3, 36]:

$$\alpha_{\text{s}}=c_{1}d^{3}f^{4},\left(\lambda >>d\right)$$ (4)

If the matrix of the modeling core has the same attenuation properties with that of the real core, we can obtain:

$$\frac{\alpha_{\text{s}}}{\alpha_{\text{mod}-\text{s}}}=\frac{d^{3}f^{4}}{d_{\text{mod}}^{3}f_{\text{mod}}^{4}}=\frac{f}{f_{\text{mod}}}=\frac{d_{\text{mod}}}{d}$$ (5)

Therefore, the ratio of the real scattering attenuation coefficient to the modeling scattering attenuation coefficient is equal to the scale factor when the modeling core is geometrically similar to the real core, and the matrix of the modeling core has the same properties (elastic moduli and density) with the real core.

### Geometrical model of porous rocks (2-Dimentional)

We need to build geometrical models of rocks with different pore structures to carry out pore-scale modeling. We also need to mesh the pore with certain numbers of elements to obtain reliable numerical results. However, the pore shape in naturally occurring rocks is irregular, and the mean pore size varies around several tens of microns. If we directly develop the geometrical model based on digital images of real cores without simplification and processing, two problems would probably occur. One is that the size of meshed elements would be much smaller than pores, which will lead to bad element quality, heavy calculation burden and even failure in convergence for a transient problem modeling. The other problem is a failure in meshing due to irregular pore shapes, such as sharp corners and intersection points. Therefore, we have to make some simplifications when we build the geometrical model:

1. Different pore shapes are modeled by regular elliptical pores with different major axis and aspect ratios (*AR*, the ratio of the minor axis to the major axis). Elliptical pores are arbitrarily orientated and randomly distributed in the matrix.
2. We magnify the pore size by a scale factor according to the similarity principle. This kind of magnification has the same theoretical basis with the physical modeling of reflection seismic exploration. They are both based on the similarity principle of wave theory.

**Fig 1:**
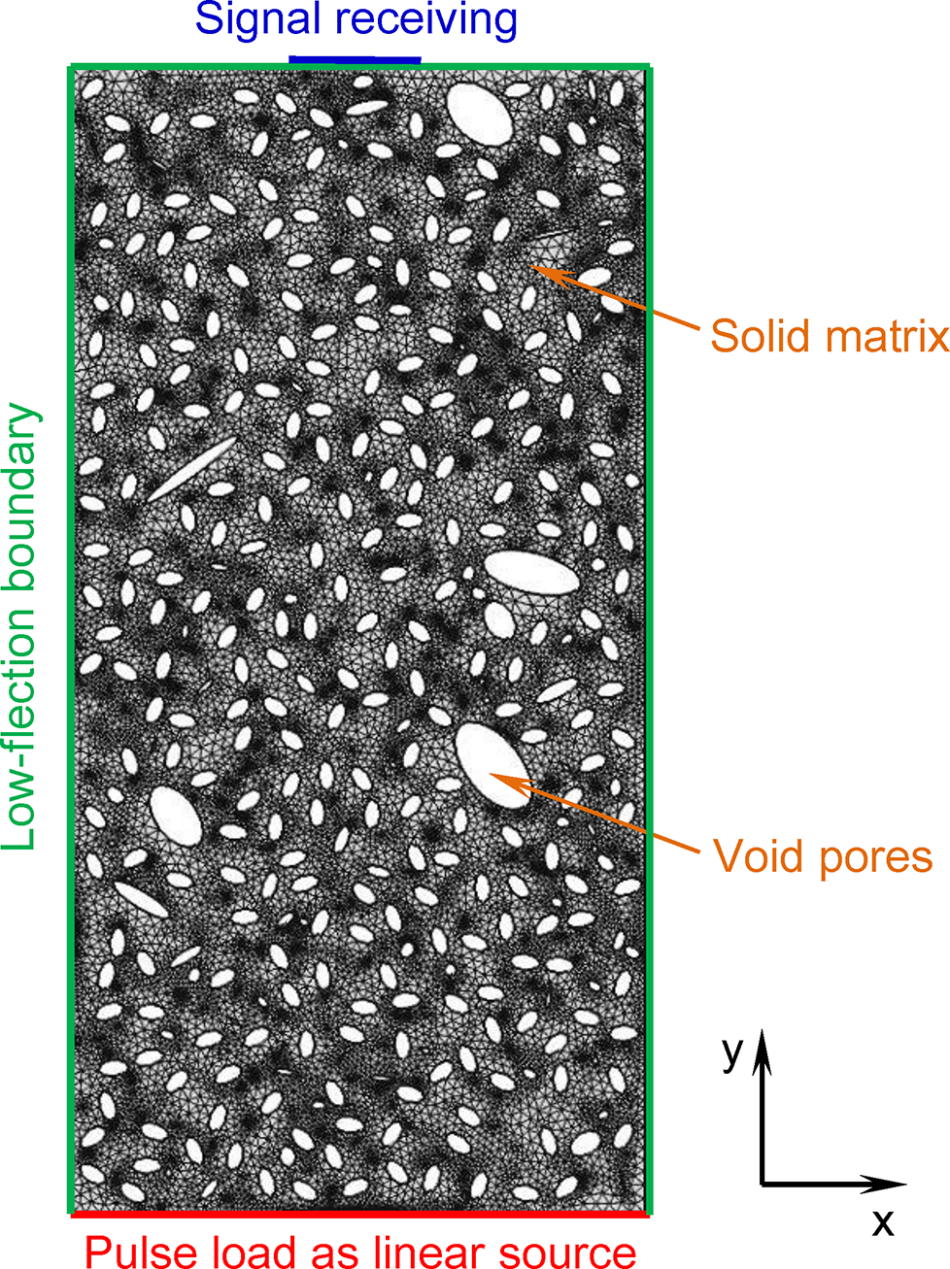
Pore-scale numerical model of elastic wave propagation in porous media. Elliptical pores with different sizes and aspect ratios are arbitrarily orientated and randomly distributed in the matrix. A pulse load is added to one side of the modeling core as a source. The other three sides are low-reflection boundaries. Receiving points are equidistantly located on the center part of the side opposite to the source.

Pores in our geometrical models do not intersect. There is because, for dry rocks, the effect of pore interconnection on the elastic wave attenuation is very small and can be neglected, because the viscosity and compressibility of air is very small compared to those of fluids, such as water and oil. This has been confirmed by the experimental observation that dry rocks (with certain pore structure) do not show velocity dispersion at different frequencies. The method of drawing geometrical models containing pores is fully described in one of our published papers (reference [37]). A typical geometrical model of porous rocks developed by this method is shown in Fig 1. The geometrical model can be meshed by most finite element method (FEM) software.

### Model description

The modeling core is made up of solid matrix and pores (Fig 1). The solid matrix is a homogeneous, isotropic and linear elastic material, which has the same elastic moduli and density as real cores. The wave equation controlling the solid matrix domain is Eq 1. In order to gain the scattering attenuation of dry rocks, the pores in the modeling core are saturated with air, the density and elastic moduli of which can be neglected compared with those of the solid matrix. The boundaries of pores are set to be free because air has a much larger compressibility than the matrix.

An excitation pulse signal with frequency *f*_mod_ is applied on one side of the modeling core in *y*-direction as a source of P- wave pulse:

$$u_{y}\left(x,0\right)=\left\{ \begin{array}{l} A_{0}\;\text{sin}\left(2\pi f_{\text{mod}}\cdot t\right),t\leq \frac{1}{f_{\text{mod}}}\\ 0,t>\frac{1}{f_{\text{mod}}} \end{array}\right.$$ (6)

The other three sides are treated to be low reflection in order to eliminate the influence of reflection from modeled-domain boundaries. The low reflection boundary takes the material data from the adjacent domain in an attempt to create a perfect impedance match for both compressional wave and shear wave, so that waves can pass out from the modeled domain without reflection [38]:

$$\sigma \cdot n=-\rho c_{\text{p}}\left(\frac{\partial u}{\partial t}\cdot n\right)n-\rho c_{\text{s}}\left(\frac{\partial u}{\partial t}\cdot \tau \right)\tau$$ (7)

Where ***τ*** is the unit tangential vector at the boundary, and *c*_p_ and *c*_s_ are the speeds of the compressional and shear waves in adjacent domains.

The initial condition of the numerical modeling is:

$$\left\{ \begin{array}{l} u\left(x,y\right)|{}_{t=0}=0\\ \frac{\partial u\left(x,y\right)}{\partial t}|{}_{t=0}=0 \end{array}\right.$$ (8)

We set ten equidistant receiving points at the center part of the side opposite to the source. Numerical calculation for the definite problem described by Eqs 1 and 6–8 are carried out by an FEM software, such as ANSYS, ABAQUS, and COMSOL. We use generalized minimal residual (GMRES) method combined with a preconditioning method of symmetric successive over-relaxation (SSOR) to solve the linear equations with speed-up convergence. To control the numerical error within an acceptable range, the maximum size of the mesh grids is 1/(10–15) of the wavelength, and the maximum time step is 1/(50–75) of the pulse source period. After the displacement ***u***(*x*, *y*) at every time step is calculated, the displacement at the receiving points can be extracted as a function of time. We take the arithmetic-averaged signal from ten receiving points as the received signal.

### Data processing

We use a linear pulse source and low-reflection boundaries in our numerical modeling (Fig 1). Therefore, the P-wave can be treated as a plane wave when it propagates through the modeling core. Geometry attenuation caused by energy dissipation can be neglected for plane waves because the wave fronts do not expand during wave propagation. Generally, the amplitude of plane wave attenuates exponentially with its propagation distance [3]. Hence, the attenuation coefficient of our modeling core can be calculated by:

$$\alpha_{\text{mod}}=\frac{\text{ln}\;A_{0}-\text{ln}\;A}{l}$$ (9)

where *A*_0_ is the amplitude of the source, *A* is the amplitude of the received signal, and *l* is the propagation distance which is equal to the core length here. We take a same size blank core without pores as a reference. The attenuation coefficient of the reference core is:

$$\alpha_{\text{mod-ref}}=\frac{\text{ln}\;A_{0}-\text{ln}\;A_{\text{ref}}}{l}$$ (10)

where *A*_ref_ is the amplitude of the received signal from the reference core. The boundary conditions and parameters of the reference core are the same as those of modeling cores. Therefore, the scattering attenuation solely caused by dry pores can be quantified by the attenuation coefficient difference between the modeling core and the corresponding reference core. That is:

$$\alpha_{\text{mod}-\text{s}}=\alpha_{\text{mod}}-\alpha_{\text{mod}-\text{ref}}=\frac{\text{ln}\;A_{\text{ref}}-\text{ln}\;A}{l}$$ (11)

The received signal is greatly deformed and no longer a sine wave because of attenuation after transmitting the modeled domain. We take the first peak of the received signal as its amplitude to calculate the attenuation coefficient.

### Repeatability of numerical modeling

**Fig 2:**
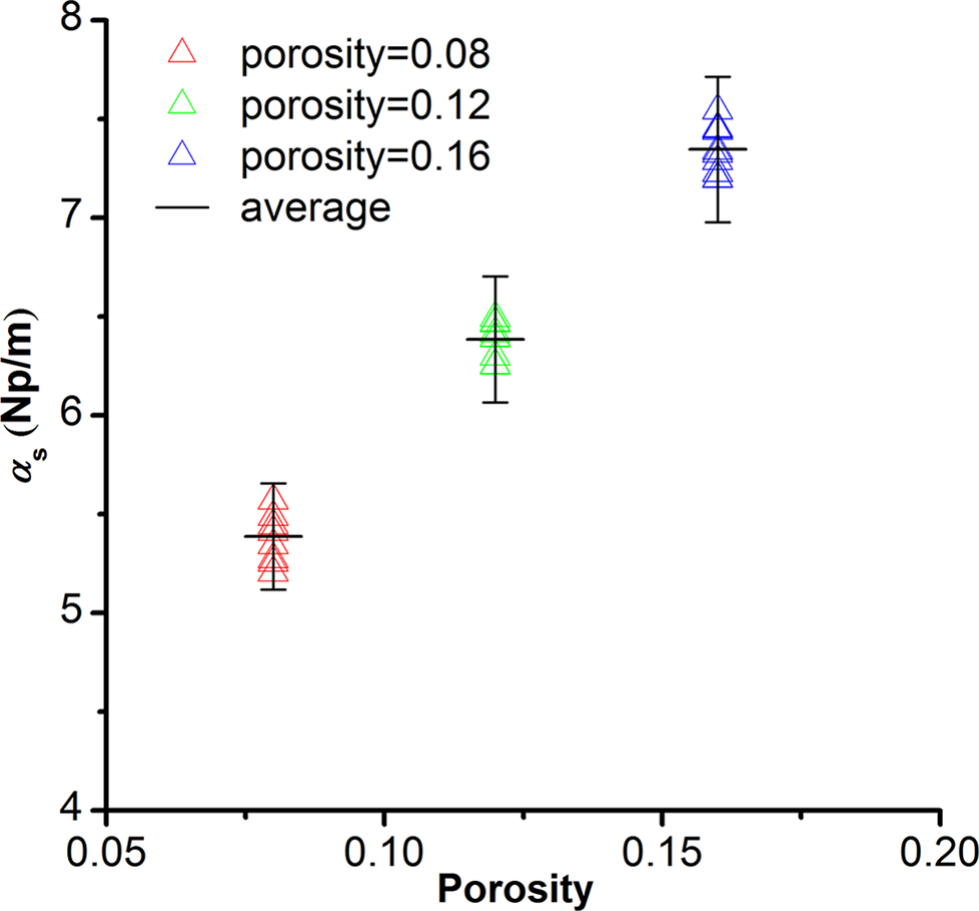
Effects of elliptical pore distribution on the modeled scattering attenuation. We calculate 10 models with randomly distributed elliptical pores at the porosity of 8%, 12%, and 16%, respectively. The pore aspect ratio and pore size in these models are constant: *AR*mod = 0.55, *d*mod = 3 mm. The modeling frequency is 0.1MHz. The error bar is 5% of the average modeled scattering attenuation coefficient.

Elliptical pores are randomly distributed in the geometrical model, leading to that two different models with the same pore structure parameters but different pore distributions may have different modeling results. The effect of pore distribution on the modeling result can be estimated through repeated modeling. We made ten times repeated modelings at the porosity of 8%, 12%, and 16% respectively. The pore size, pore aspect ratio, and modeling frequency are kept constant (*d*_mod_ = 3mm, *AR* = 0.55, *f*_mod_ = 0.1MHz). The modeling results are shown in Fig 2.

Variation in the modeled scattering attenuation decreases as the porosity increases (Fig 2). This indicates that although random distribution of pores has a lessened effect on scattering attenuation at a higher porosity, the effects of pore distribution on the modeling results are always less than 5% of the modeled scattering attenuation. The modeling results are stable and well repeatable for any single porosity point and the overall trend.

### Numerical modeling design

The pore structure of carbonate rocks is complex, so the pore structure effects on P-wave scattering attenuation in dry carbonates are significant and require more attention in the field data analysis. Therefore, we take carbonate rocks as an example to address this issue in the following. The values of parameters used in our modeling are shown in Table 1, which are calculated based on the similarity principle and pore structure data from digital images of 120 carbonate cores [39]. The scale factor is equal to ten, which is proper to simulate the ultrasonic pulse-transmission measurements in the lab. Under this condition, the modeling scattering attenuation coefficient is one tenth of the ultrasonic measurements according to Eq 5.

**Table 1:** Scale factors and parameters used in the numerical modeling.

Parameters	Ultrasonic test in lab	Scale factors	Numerical modeling
**Matrix properties** ^a^(95%Calcite+5%Clay)	K = 71.9GPa, *μ* = 29.6GPa, *ρ* = 2.70g/cm^3^,	1:1	*K*_mod_ = 71.9GPa, *μ*_mod_ = 29.6GPa, *ρ*_mod_ = 2.70g/cm^3^,
**Frequency**	*f* = 0.1-2MHz	10:1	*f*_mod_ = 0.01–0.2MHz
**Pore size**	*d* = 10–10^3^um	1:10	*d*_mod_ = 0.1-10mm
**Aspect ratio**	*AR* = 0.01–1	1:1	*AR*_mod_ = 0.01–1
**Porosity**	*φ* = 0–0.4	1:1	*φ*_mod_ = 0–0.4
**Scattering attenuation coefficient**	*α*_s_	10:1	*α*_mod-s_

^a^ The effective elastic moduli of the solid matrix are calculated by Hill’s average according to the elastic moduli of its mineral composite [36]. And the effective density is calculated by volume average.

For a 2-dimentional domain (*S*_0_) containing *N* elliptical pores with the same major axis (*d*) and aspect ratio (*AR*), porosity (*φ*) can be written as:

$$\phi =\frac{\pi }{4}\frac{N}{S_{0}}d^{2}\;AR$$ (12)

Here we define *ε* = *N*/*S*_0_ as pore density which represents the number of pores per unit area. For an individual pore, it can be described by pore size and pore aspect ratio. For the whole sample, it still needs porosity or pore density to show the amount of pores. Only three of these four pore structure parameters (porosity, pore size, pore density, and pore aspect ratio) are independent. In this study, we use pore density, pore aspect ratio, and pore size as key parameters to describe the pore structure. In order to investigate the pore structure effects on P-wave scattering attenuation, we build series of geometrical models with different pore structures according to the controlling variable principle. The controlling variable principle means there should be only one varying factor when the properties of interest are compared; therefore, the changes of the interested properties can be attributed to only one varying factor. For example, when we study the effects of pore size on P-wave scattering attenuation, we build geometrical models with varying pore size but constant pore density and pore aspect ratio. We attribute the changes in modeled P-wave scattering attenuation to the varying pore size. In the following, we will firstly discuss the effects of pore size, pore density, and pore aspect ratio on scattering attenuation, respectively, at certain porosity. Subsequently, we will analyze the scattering attenuation variation with porosity to show the combined effects of pore size, pore density, and pore aspect ratio.

## Results and Discussion

### Pore size effect on scattering attenuation

**Fig 3:**
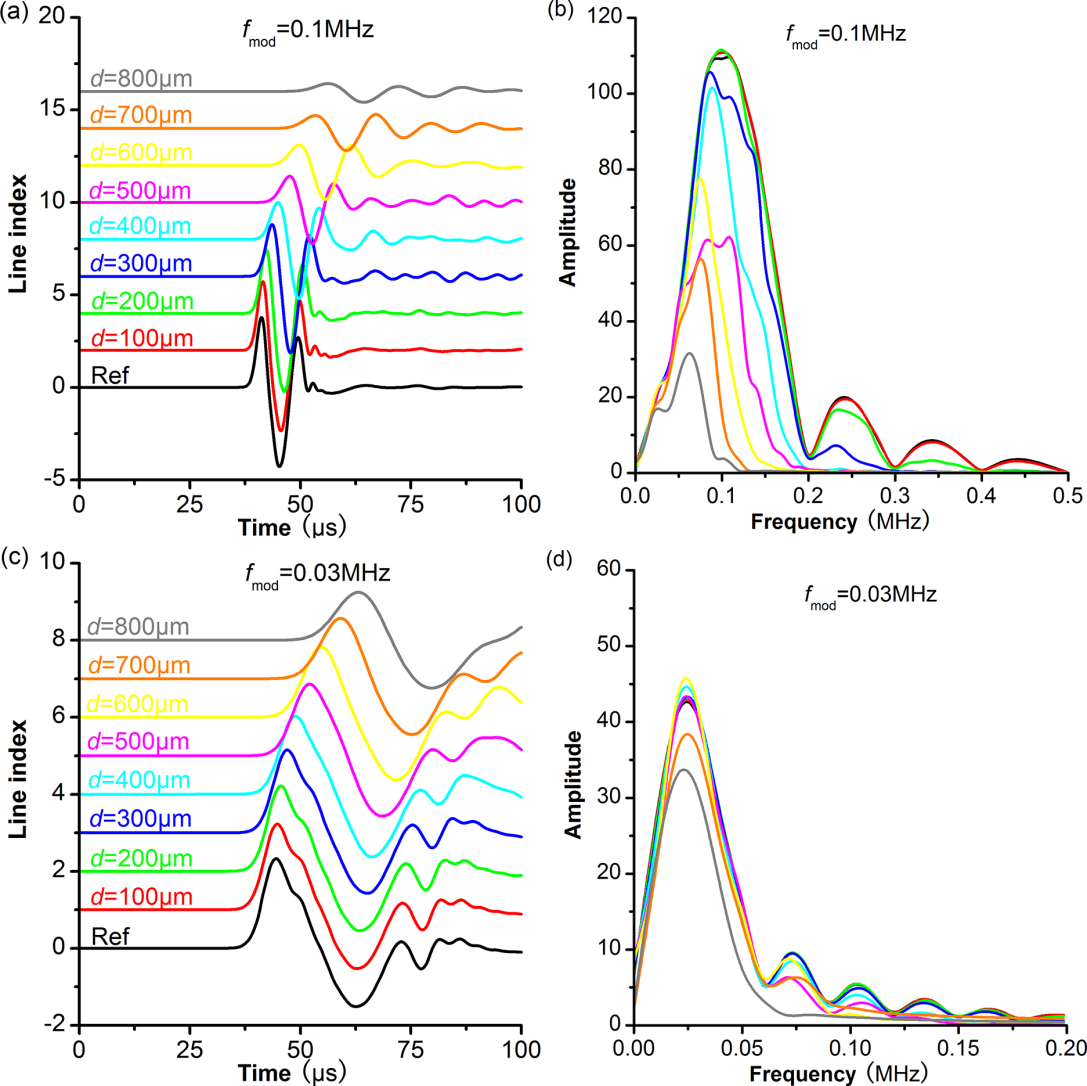
The received signals and corresponding frequency spectrums from models with different pore sizes. Different colors are used to discriminate signals from the reference core and the modeling cores with different pore sizes. The pore aspect ratio and pore number in these models are constant: *AR*_mod_ = 0.55, *N* = 300. The source frequency (*f*_mod_) is shown at the top of the figure. (a) and (c) are received signals; (b) and (d) are the corresponding frequency spectrums.

In order to analyze the effects of pore size on P-wave scattering attenuation, we build models with varying pore sizes at source frequency of 0.1MHz and 0.03MHz, respectively. For all these models, we keep pore aspect ratio as 0.55, and pore number as 300. The received signals and corresponding frequency spectrums are shown in Fig 3.

When the source frequency is 0.1MHz, the wavelength of the received signal is about 60-80mm, and the ratio of wavelength to pore size (*λ/d*) ranges 9–60. With an increase in the pore size, the amplitudes of received signals significantly decrease (Fig 3A), and the main frequencies shift to much lower values (Fig 3B). This indicates that the P-wave attenuates greatly after propagating through cores with larger pores. When the source frequency is 0.03MHz, the wavelength of the received signal is 140-210mm, and the ratio of wavelength to pore size (*λ/d*) is 18–210. With an increase in the pore size, the amplitudes of received signals decrease slightly (Fig 3C), and the main frequencies do not show noticeable shift (Fig 3D). Therefore, the P-wave attenuation at 0.03MHz is much weaker than that at 0.1MHz. The scattering effect of larger pores is stronger at higher frequencies, which results in higher attenuation.

**Fig 4:**
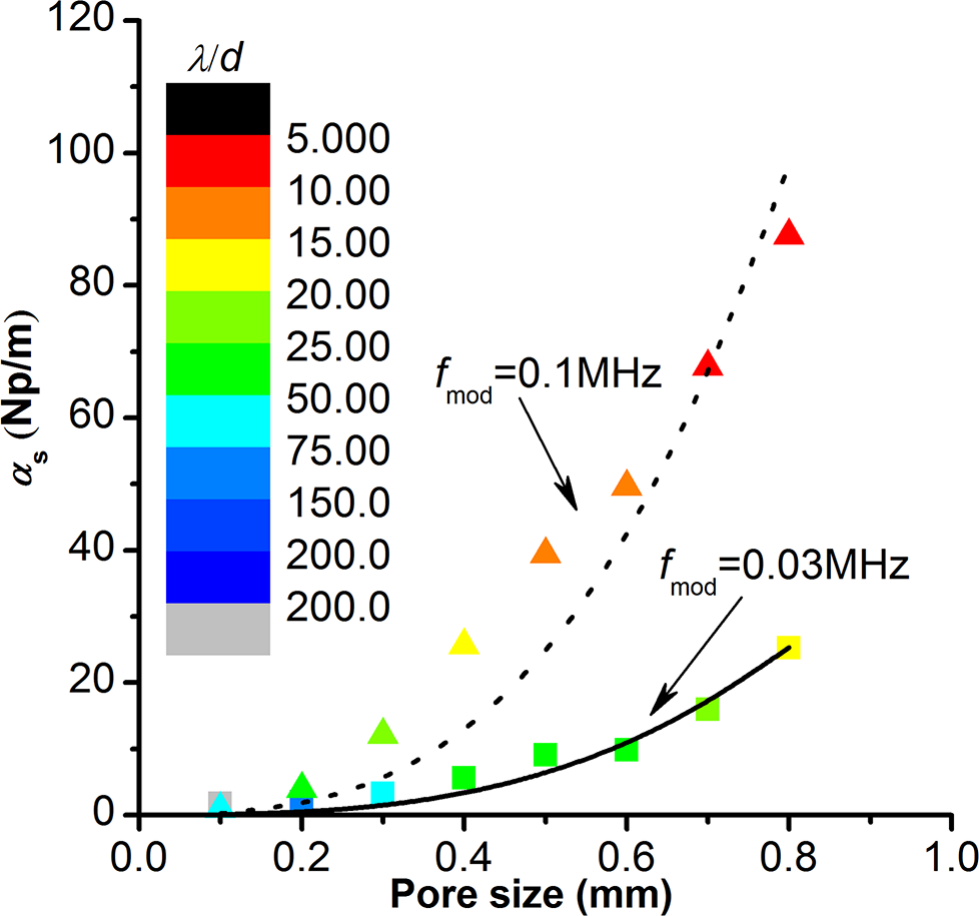
Influence of pore size on the scattering attenuation coefficient. Data points are from the numerical modeling: triangular points for the source frequency at 0.1MHz, square points for the source frequency at 0.03MHz. The color of data points indicates the ratio of wavelength to pore size. The dotted line and the solid line are fitted by Eq 4 respectively. The pore aspect ratio and pore number in these models are constant: *AR*_mod_ = 0.55, *N* = 300.

We calculate the scattering attenuation coefficients (Fig 4) according to Eq 11 based on numerical results. In Fig 4, data points are from numerical modeling, and the color of the data point represents its corresponding value of *λ/d*. The solid and dotted lines are fitted by Eq 4 which indicates that the scattering attenuation coefficient is proportional to the third power of the pore size when the wavelength is much larger than the pore size (*λ*>>*d*). But the meaning of *λ*>>*d* is left ambiguous. When *λ/d* is larger than 20, the numerical modeling results are consistent with the trend described by Eq 4. However, the scattering attenuation coefficient is larger than that estimated by Eq 4 when *λ/d* is smaller than 15. Therefore, a conservative estimate of *λ*>>*d* should be *λ/d* > 15 from the perspective of scattering attenuations, which means the composite including heterogeneous inclusions can be treated as homogenous effective media to analyze the P-wave scattering attenuation when *λ/d* > 15.

### Pore density effect on scattering attenuation

**Fig 5:**
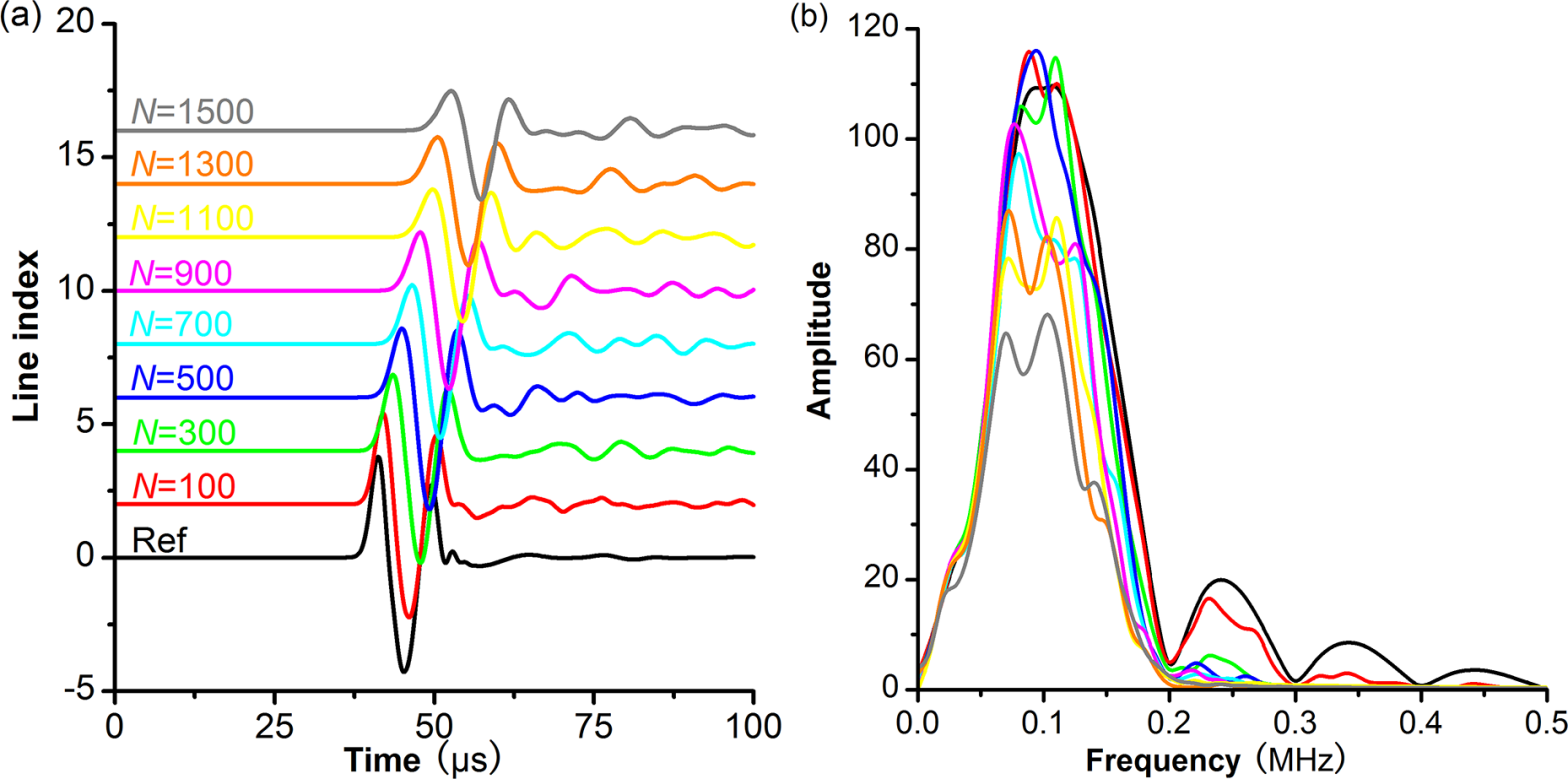
The received signals and corresponding frequency spectrums from models with different pore densities. Different colors are used to discriminate signals from the reference core and the modeling cores with different pore numbers. The modeled domain is fixed, so different pore numbers result in different pore densities. The pore aspect ratio and pore size in these models are constant: *AR*_mod_ = 0.55, *d*_mod_ = 3mm.

We built a variety of models keeping pore aspect ratio, pore size, and source frequency unchanged (*AR* = 0.55, *d*_mod_ = 3mm, and *f*_mod_ = 0.1MHz) whereas the pore number varies to investigate the pore density effects on scattering attenuation. The size of the model domain (width×length) is 0.125m ×0.25m.The wavelength is about 50-63mm, and the ratio of wavelength to pore size is 17–21. Therefore, it is safe to regard as *λ*>>*d*. The received signals and corresponding frequency spectrums of models with different pore densities are shown in Fig 5.

**Fig 6:**
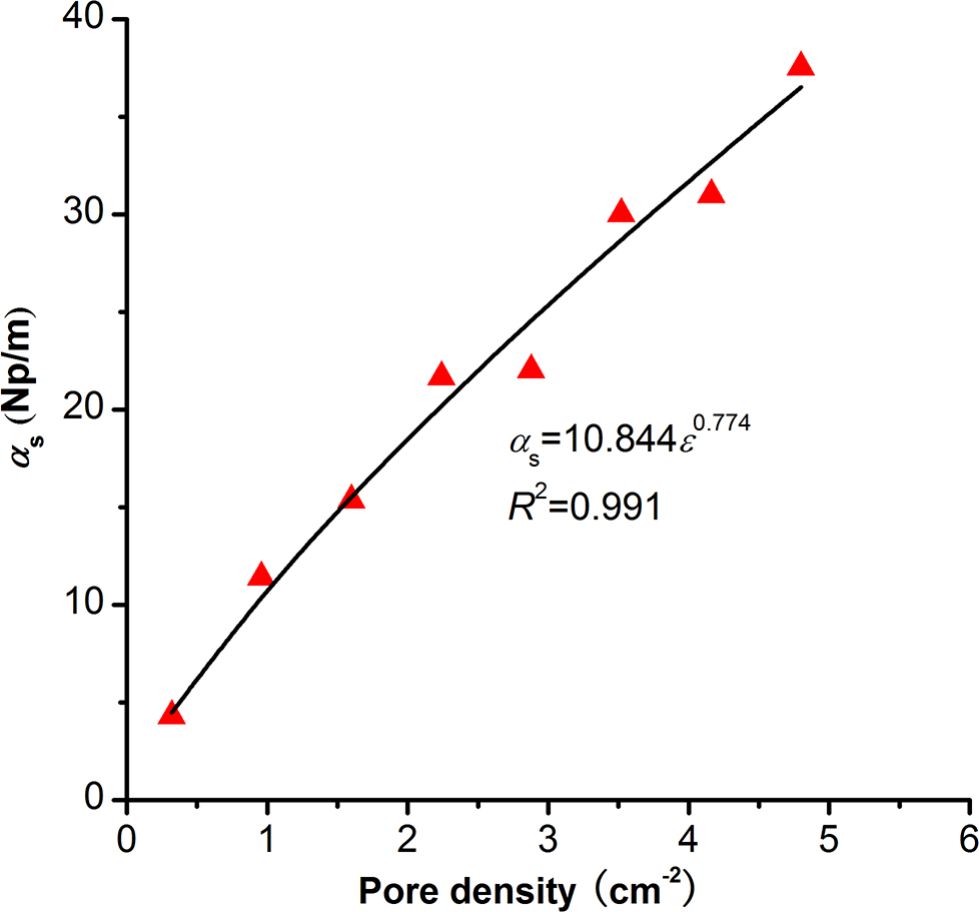
Influence of pore density on the scattering attenuation coefficient. Data points are from the numerical modeling at *f*_mod_ = 0.1MHz. The solid line is fitted by Eq 13. The pore aspect ratio and pore size in these models are constant: *AR*_mod_ = 0.55, *d*_mod_ = 3mm.

With an increase in pore density, the amplitudes of received signals greatly decrease (Fig 5A), and the main frequencies slightly shift to lower values (Fig 5B), which indicate that the P-wave attenuation increases as the pore density increases. This is because there are more reflections and diffractions when the pore density increases. An increase in reflections and diffractions results in higher scattering attenuation. We calculate the scattering attenuation coefficients according to Eq 11 based on numerical results (Fig 6). Under long wavelength condition, the scattering attenuation coefficient increases a power function as pore density increases:

$$\alpha_{\text{s}}=a_{1}\varepsilon^{a_{2}}$$ (13)

Where, *ε* is the pore density, *a*_1_ and *a*_2_ are fitting parameters related to the pore shape.

### Pore aspect ratio effect on scattering attenuation

**Fig 7:**
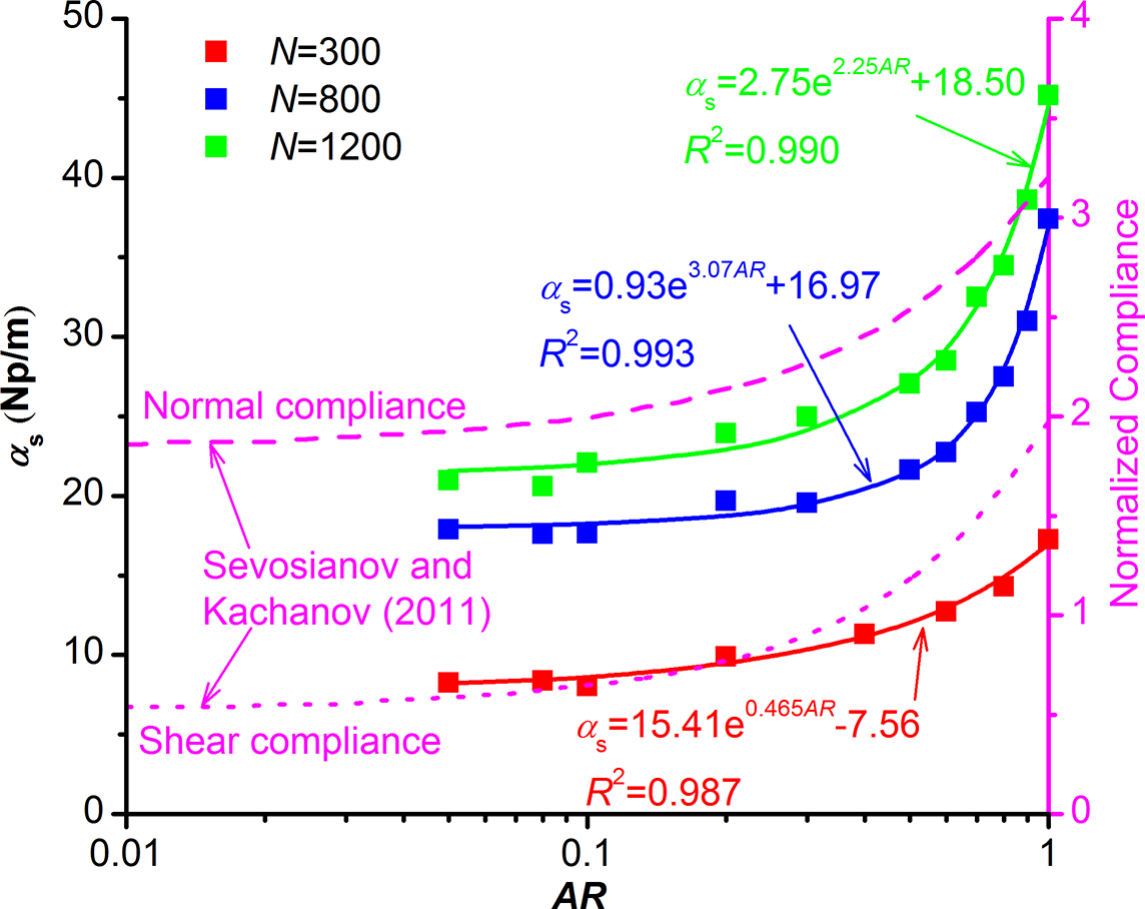
Influence of pore aspect ratio on the scattering attenuation coefficient. Data points are from the numerical modeling with varying pore aspect ratio at pore number of 300, 800, and 1200 respectively. The solid line is fitted by Eq 14. The pore number, pore size, and source frequency in these models are constant: *N* = 300, *d*_mod_ = 3mm, *f*_mod_ = 0.1MHz. The magenta dashed lines are from Sevosianov and Kachanov [22].

We usually examine the effect of pore aspect ratio on the physical properties of porous rocks by keeping the porosity unchanged. However, this implies that the pore size and (or) pore density must decrease as the aspect ratio increases. The proper way to examine the influence of aspect ratio on P-wave scattering attenuation is to keep pore size and pore density, not porosity, fixed as the aspect ratio changes[40]. We build three series of models with a varying *AR* at pore number of 300, 800, and 1200 respectively. In each series, the pore size and the source frequency stay constant (*d*_mod_ = 3mm, *f*_mod_ = 0.1MHz). The wavelength of received signals is about 48-61mm, and the ratio of wavelength to pore size is 16–20. The scattering attenuation coefficient increases exponentially with the increase of aspect ratio at the same pore number (Fig 7). Their relationship can be written as:

$$\alpha_{\text{s}}=p_{1}+p_{2}e^{p_{3}AR}$$ (14)

where *p*_1_, *p*_2_, and *p*_3_ are fitting parameters. The solid lines in Fig 7 based on Eq 14 fits these modeling data quite well. As shown in Fig 7, when the aspect ratio is smaller than 0.2, the scattering attenuation coefficient is insensitive to the change of aspect ratio; but as the aspect ratio increases, especially after *AR*>0.5, the scattering attenuation coefficient increases rapidly. This is because an increase in aspect ratio results in higher porosity, which makes the propagation path of P-wave more circuitous and leads to higher scattering level. The variation trend of scattering attenuation with *AR* is similar to that of normalized compliances with *AR* which is obtained by Sevosianov and Kachanov [22] through analytical approaches. This indicates that the scattering attenuation should be related to the rock’s elastic compliances.

### Porosity effect on scattering attenuation

Experiment results [23–24] indicate that the elastic wave attenuation increases as the porosity increases. However, the porosity is controlled by pore density, pore size, and pore aspect ratio according to Eq 12. We have discussed the effects of these three factors on scattering attenuation in the above parts, and found that their contributions to scattering attenuation coefficients are not the same. Therefore, it is probable that pore structure can explain the variation of scattering attenuation at the same porosity, and to some extent responsible for the wide scattering of attenuation-porosity cross-plots of rocks with complex pore structures.

**Fig 8:**
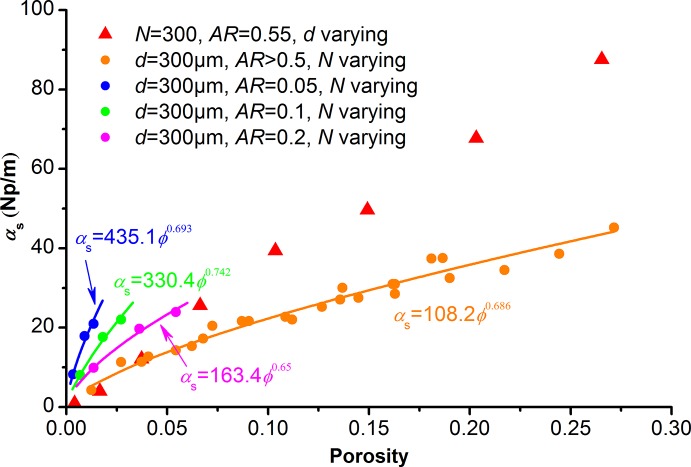
Influence of porosity on the scattering attenuation coefficient. Data points are from the numerical modeling at *f*_mod_ = 0.1MHz. The triangular points are from models with varying pore size. The solid circle points are from models with varying pore number and pore aspect ratio, and the corresponding *λ/d* is larger than 15. The solid lines are fitted by Eq 15.

We plot all modeled P-wave scattering attenuation coefficients at *f*_mod_ = 0.1MHz in one figure with porosity as the horizontal axis (Fig 8). The circle points in Fig 8 have constant pore size (*λ/d*>15). The triangular points represent results of different pore sizes at constant pore aspect ratio. For rocks with complex pore structure, the variation of scattering attenuation at the same porosity is mainly caused by changes in pore size and pore shape (aspect ratio). Rocks with smaller aspect ratio and (or) larger pore size have stronger scattering attenuation at the same porosity. When the pore aspect ratio is unchanged, the scattering attenuation coefficient increases as a power function as the porosity increases under long wavelength (*λ/d*>15):

$$\alpha_{\text{s}}=b_{1}\phi^{b_{2}}$$ (15)

where *b*_1_ and *b*_2_ are fitting parameters related to the pore aspect ratio. It is interesting that the fitting parameters (*b*_1_, *b*_2_) are sensitive to aspect ratio when *AR* is smaller than 0.2, and seem less dependent on aspect ratio when *AR* is larger than 0.5. This means that pore size is dominantly responsible for the variation of scattering attenuation at the same porosity when *AR* is larger than 0.5.

## Conclusions

We investigated the pore structure effects on P-wave scattering attenuation in dry rocks by pore-scale modeling based on the wave theory and similarity principle. Our modeling results indicate that pore size, pore shape (aspect ratio), and pore density are important factors influencing P-wave scattering attenuation in porous rocks, and can explain the variation of scattering attenuation at the same porosity. From the perspective of scattering attenuation, porous rocks can safely suit to the long wavelength assumption when the ratio of wavelength to pore size is larger than 15. At long wavelength, the scattering attenuation coefficient increases as a power function as the pore density increases, and it increases exponentially with an increase in aspect ratio. For a certain porosity, rocks with smaller aspect ratio and (or) larger pore size have stronger scattering attenuation. When the pore aspect ratio is larger than 0.5, the variation of scattering attenuation at the same porosity is dominantly caused by pore size and almost independent of the pore aspect ratio.

## Supporting Information

S1 Table Data of modeling repeatability test. (XLSX)

S2 Table Data of signals and corresponding frequency spectrums with different pore sizes. (XLSX)

S3 Table Data of scattering attenuation coefficients for different pore sizes. (XLSX)

S4 Table Data of signals and corresponding frequency spectrums with different pore densities. (XLSX)

S5 Table Data of scattering attenuation coefficients for different pore densities. (XLSX)

S6 Table Data of scattering attenuation coefficients for different pore aspect ratios. (XLSX)

S7 Table Data of scattering attenuation coefficients for different porosities. (XLSX)
